# Analysis of the Light Propagation Model of the Optical Voltage Sensor for Suppressing Unreciprocal Errors

**DOI:** 10.3390/s17010085

**Published:** 2017-01-03

**Authors:** Hui Li, Zhida Fu, Liying Liu, Zhili Lin, Wei Deng, Lishuang Feng

**Affiliations:** 1 Key Laboratory of Precision Opto-mechatronics Technology, Ministry of Education, School of Instrumentation Science and Opto-electronics Engineering, Beihang University, Beijing 100191, China; fuzhida@buaa.edu.cn (Z.F.); xiaocuter@hotmail.com (L.L.); deng1517101@buaa.edu.cn (W.D.); fenglishuang@buaa.edu.cn (L.F.); 2 Fujian Key Laboratory of Light Propagation and Transformation, College of Information Science and Engineering, Huaqiao University, Xiamen 361021, China; zllin2008@gmail.com

**Keywords:** optical voltage sensor, measurement accuracy, temperature stability

## Abstract

An improved temperature-insensitive optical voltage sensor (OVS) with a reciprocal dual-crystal sensing method is proposed. The inducing principle of OVS reciprocity degradation is expounded by taking the different temperature fields of two crystals and the axis-errors of optical components into consideration. The key parameters pertaining to the system reciprocity degeneration in the dual-crystal sensing unit are investigated in order to optimize the optical sensing model based on the Maxwell's electromagnetic theory. The influencing principle of axis-angle errors on the system nonlinearity in the Pockels phase transfer unit is analyzed. Moreover, a novel axis-angle compensation method is proposed to improve the OVS measurement precision according to the simulation results. The experiment results show that the measurement precision of OVS is superior to ±0.2% in the temperature range from −40 °C to +60 °C, which demonstrates the excellent temperature stability of the designed voltage sensing system.

## 1. Introduction

More reliable, economical and effective smart grids have become an important development trend in electric power grids, where voltage sensors are the key components for metering electric energy and relay protection in electric power systems [1,2]. Optical voltage sensors (OVSs) are a type of voltage sensors that are more suitable for application in smart grids due to their advantages over the conventional counterparts [3,4], including inherent electrical insulation, lightweight and wide bandwidth. 

In the past few decades, a number of studies have been carried out to design and optimize the optical sensing methods of OVS in order to improve the measurement precision. The potential optical effects that can be used for voltage sensing mainly include the Pockels effect, converse piezoelectric effect, fiber Bragg grating (FBG) and Kerr effect, etc. Recently, a high-voltage sensor system [5] using a PZT piezoelectric crystal as a transducer and a FBG as a strain measuring sensor that successfully met the demands IEC60044-5 for 0.2 level measurement was proposed. Ribeiro also developed a novel optimization algorithm to improve the sensitivity on demodulation of the FBG-based optical voltage sensor for 13.8 kV class [6]. FBG-based optical sensors may measure voltage or current in a high voltage line. However, OVSs based on the Pockels effect of Bi_4_Ge_3_O_12_ (BGO) crystals have become the main research focus in the voltage sensing field [7,8]. Lee proposed a compensation method with double light paths to eliminate the unwanted influence of birefringence on OVS [9]. To directly measure the high voltage, Santos et al. designed a prototype of OVS with the Pockels device in a multi-segmented arrangement [10]. Chavez et al. proposed a measuring method for high voltages by using several electric field sensors and adopting a quadrature method [11]. Recently, Kumada et al. introduced a two-wavelength dual laser system with 8 in-line BGO crystals to measure the high voltage [12]. Li et al. utilized twice total reflection of incident light in a Fresnel rhomb BGO crystal to replace a 1/4 wave plate that generates a π/2 optical phase bias [13]. Chu et al. presented an integrated optical device consisting of a coupler, polarizer, phase modulator and polarization converter to control the operating bias point of optical voltage transducers [14]. Then, the integrated optical device was applied in a reflection type optical voltage sensing to optimize the temperature dependence of OVS [15]. A closed-loop detection system of OVS was proposed to reduce the gain drift of a forward channel caused by the temperature changes [16] and a closed-loop algorithm of OVS was designed to improve the detection precision of low voltage considering the cross coupling of two closed-loops [17]. All of these studies contributed to the progress of research on OVS. However, the temperature stability performance of optical sensing method is still the main influence factor for the application of OVS in a smart grid.

In our previous work, a dual-crystal sensing scheme of OVS was proposed to theoretically alleviate the temperature sensitivity [18]. However, system reciprocity degeneration is unavoidable due to the influence of various factors in the manufacturing process, particularly the undesirable axis-angle errors of optical components and the uneven temperature fields in the two crystals. In this work, the inducing principle of OVS reciprocity degradation is investigated and some methods for suppressing unreciprocal errors are proposed to improve the measurement precision of OVS. Firstly, the light propagation model in the dual-crystal sensing unit is analyzed based on Maxwell's electromagnetic theory. For the stress distribution symmetry, the key parameters of the sensing unit are explored and the sensing unit structure is designed to ensure dual-crystal reciprocity. Moreover, the influencing principle of axis-angle errors on the nonlinearity between the OVS input and output in the Pockels phase transfer unit is analyzed. Then, a novel axis-angle compensation method is proposed to neutralize unavoidable axis-angle errors of optical components in the manufacturing process of OVS, which is important for the high sensitivity of measurement. Finally, experiments are conducted to demonstrate the validity and effectiveness of the proposed sensing methods.

## 2. Theoretic Modeling and Error Analyses

The optical structure of OVS includes the Pockels phase transfer unit and the voltage sensing unit. In the Pockels phase transfer unit, the light emitted from the super luminescent diode (SLD) passes through the single mode (SM) circulator and gets polarized by the polarizer. Then, the light is split into two orthogonal linear-polarized lights by a 45° splice before propagating into the optical phase modulator (OPM). The reciprocity of the voltage sensing unit is realized by the two orthogonal polarization modes swapped in the dual-crystal that neutralize the temperature errors. The lights retrace the original path after being reflected by the mirror of crystal B, then interfere at the polarizer with a double Pockels phase, and finally enter the PIN-FET.

**Figure 1:**
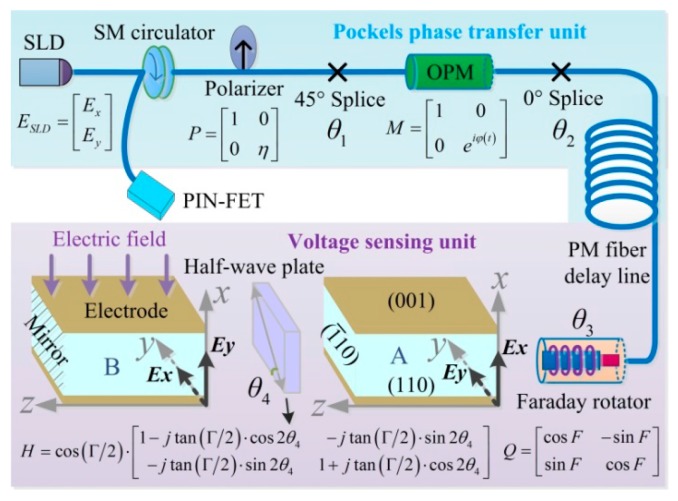
The working principle of the proposed OVS.

The working principle of the proposed OVS is shown in Figure 1, where voltage is only applied on crystal B. The axes *x*, *y*, *z* are the voltage-induced principal axes of refractive index of the BGO crystal, and *Ex* and *Ey* are the oscillating directions of the two orthogonal linear-polarized lights, respectively. The optical parameter *η* is the polarizer’s polarization extinction coefficient, *θ*_1_ and *θ*_2_ are, respectively, the practical welding angles of the 45° splice point and 0° splice point, *φ*(t) is the modulation phase applied on OPM, *F* is the actual rotation angle of the Faraday rotator (FR), *θ*_3_ is the actual axis-angle between the FR and the BGO crystal, Γ is the actual phase delay angle of the half-wave plate, and *θ*_4_ is the actual axis-angle between the half-wave plate and the BGO crystal.

The scheme of dual-crystal optical structure is reciprocal theoretically [18]. However, the practical uncertainty of optical parameters has a negative impact on the reciprocity of the Pockels phase transfer unit. Meanwhile, the inhomogeneous temperature field of the dual-crystal structure will cause the reciprocity degradation of the dual-crystal sensing unit, which is neglected in the previous related research [18]. Thus, the model analysis of the optical voltage sensor for suppressing unreciprocal errors is of great significance in order to improve the OVS working performance.

### 2.1. Optimal Design of Dual-Crystal Unit

Firstly, the model for the voltage sensing unit is set up by taking the unideal optical parameters into consideration under multiple physical fields. The light propagation equation in the dual-crystal is given by:

(1) $$\nabla^{2}E+\omega^{2}\mu \cdot \varepsilon_{0}\{[\varepsilon^{0}]+[\Delta \varepsilon_{P}]+[\Delta \varepsilon_{T}]+[\Delta \varepsilon_{S}]\}\cdot E=0$$

where *E* is the electric field intensity, *ω* is the angular frequency, *μ* is the permeability of the BGO crystal, and *ε*_0_ is the permittivity of the vacuum. The tensor form of the relative dielectric constant of the BGO crystal under the ideal isotropic situation is $[\varepsilon^{0}]=\left[\begin{array}{ccc} \varepsilon_{r} & 0 & 0\\ 0 & \varepsilon_{r} & 0\\ 0 & 0 & \varepsilon_{r} \end{array}\right]$, where *ε*_r_ is the relative permittivity of the crystal. However, due to the Pockels effect, thermo-optic effect and elastic-optic effect, the ensuing variations of relative dielectric tensor Δ*ε*_P_, Δ*ε*_T_ and Δ*ε*_S_ should be taken into account, respectively. 

**Figure 2:**
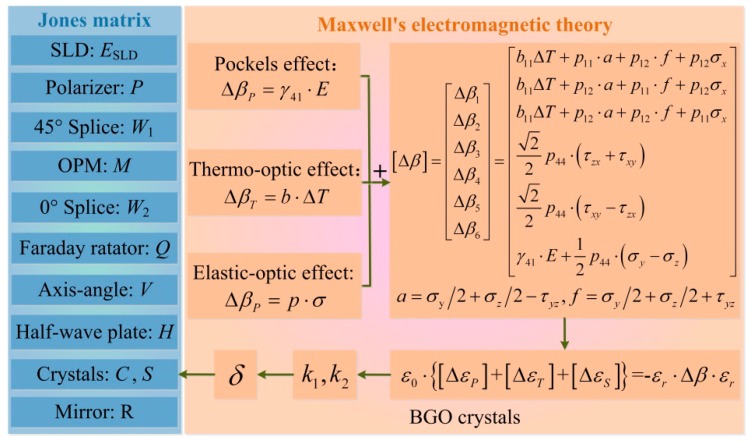
Simulation platform of OVS considering the applied electric, temperature, and stress fields and axis-angle errors.

To obtain Δ*ε*_P_, Δ*ε*_T_ and Δ*ε*_S_, we build an OVS simulation platform considering the influence of the applied electric field, the temperature field and the stress field on the crystal optical properties. As depicted in Figure 2 and mentioned previously, the total variation of the inverse dielectric tensor is [Δ*β*] = [Δ*β_P_*] + [Δ*β_T_*] + [Δ*β_S_*], where [Δ*β_P_*], [Δ*β_T_*] and [Δ*β_S_*] are caused by the Pockels effect, the thermo-optic effect and the elastic-optic effect, respectively. The quantities *b*_11_ and *p*_11_, *p*_12_, *p*_44_ are the thermo-optic coefficient and the elastic-optic coefficients of the BGO crystal, respectively. *γ*_41_ is the electro-optical coefficient of the BGO crystal. Δ*T* represents the temperature variation. σ*_x_*, σ*_y_*, σ*_z_* and τ*_xy_*, τ*_yz_*, τ*_zx_* are, respectively, the normal stresses and the shear stresses on the crystals. According to the variation in the inverse dielectric tensor, we acquire the variable quantity of the BGO crystals’ dielectric tensor.

**Figure 3:**
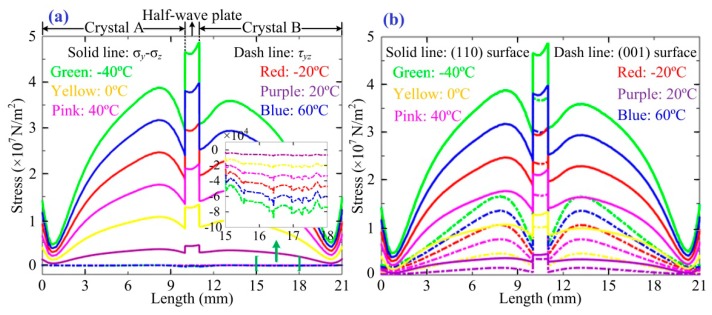
(**a**) Distributions of (*σ_y_* − *σ_z_*) and *τ_yz_* when the (110) surface of crystal is fixed on the mechanical device; (**b**) absolute values of (*σ_y_* − *σ_z_*) for the two different fixed methods under six different temperatures.

Based on the proof given in the Appendix A, we also simulate the distribution properties of (*σ_y_* − *σ_z_*) and *τ_yz_* along the light propagation path, as shown in Figure 3. We can see that (*σ_y_* − *σ_z_*) is much greater than *τ_yz_* that indicates the latter can be omitted. Meanwhile, the orders of magnitude of (*p*_12_ − *p*_11_) and *p*_44_ are 10^−13^ and 10^−12^, respectively [19], and we know that *ε_yy_* − *ε_xx_* = Δ*ε_yy_* − Δ*ε_xx_* = 2*ε*_r_^2^·*τ_yz_*(*p*_12_ − *p*_11_) and *ε_xy_*·*ε_yx_* = *ε*_r_^4^·[*γ*_41_·*E* + *p*_44_·(σ*_y_* − σ*_z_*)/2]^2^. Then, we can conclude that (*σ_y_* − *σ_z_*) is the key factor that influences the OVS temperature stability.

Based on the infinitesimal method, crystals A and B are equally divided into *q* segments, whose crystal lengths satisfy *l* = *q*·Δ*l*, where *q* is large enough. Moreover, we suppose that *σ_yc_*(*h*) and *σ_zc_*(*h*), *σ_ys_*(*h*) and *σ_zs_*(*h*) are, respectively, the normal stresses on crystal A and crystal B, where *h* = 1, 2, …, *q*. Considering that (*ε_yy_* − *ε_xx_*) << *ε_xy_ε_yx_*, we can derive that $C_{h}=\left[\begin{array}{cc} a & b\\ c & d \end{array}\right]\approx \left[\begin{array}{cc} \text{cos}\;\phi_{h} & j\;\text{sin}\;\phi_{h}\\ j\;\text{sin}\;\phi_{h} & \text{cos}\;\phi_{h} \end{array}\right]=\left[\begin{array}{cc} 1 & 0\\ 0 & e^{j\phi_{h}} \end{array}\right]$, where $\phi_{h}=\frac{\omega \sqrt{\mu \varepsilon_{0}}\varepsilon_{r}^{3/2}\Delta l}{4}\cdot p_{44}\left(\sigma_{yc}(h)-\sigma_{zc}(h)\right)$. Similar to the analysis of crystal A, the equivalent Jones matrix of each segment in crystal B is given by $S_{h}=\left[\begin{array}{cc} a^{\prime } & b^{\prime }\\ c^{\prime } & d^{\prime } \end{array}\right]=\left[\begin{array}{cc} 1 & 0\\ 0 & e^{j{\phi_{h}}^{\prime }} \end{array}\right]$, where $\phi_{h}^{\prime }=\frac{\omega \sqrt{\mu \varepsilon_{0}}\varepsilon_{r}^{3/2}\Delta l}{2}\cdot \left(\gamma_{41}\cdot E+p_{44}\cdot \frac{\sigma_{ys}(h)-\sigma_{zs}(h)}{2}\right)$.

By substituting the Jones matrix of the half-wave plate *H* and assuming that the ideal matrix is $H=-j\left[\begin{array}{cc} 0 & 1\\ 1 & 0 \end{array}\right]$, we have the equivalent Jones matrix of the dual-crystal sensing unit:

(2) $$G=C_{1}\cdot C_{2}\cdot \ldots \cdot C_{q}\cdot H\cdot S_{q}\cdot \ldots \cdot S_{2}\cdot S_{1}=-j\left[\begin{array}{cc} 0 & e^{j\cdot \sum_{h=1}^{q}\phi_{h}^{\prime }}\\ e^{j\cdot \sum_{h=1}^{q}\phi_{h}} & 0 \end{array}\right]$$

Based on the above theoretical analysis, the phase-delay caused by the Pockels effect is herewith obtained:

(3) $$\begin{array}{cc} \delta & =\sum_{h=1}^{q}\phi_{h}^{\prime }-\sum_{h=1}^{q}\phi_{h}\\ & =\frac{\omega \sqrt{\mu \varepsilon_{0}}\varepsilon_{r}^{3/2}\gamma_{41}}{2}\cdot \sum_{h=1}^{q}E_{h}\cdot \Delta l+\frac{\omega \sqrt{\mu \varepsilon_{0}}\varepsilon_{r}^{3/2}p_{44}}{2}\cdot \sum_{h=1}^{q}\frac{\left(\sigma_{ys}\left(h\right)-\sigma_{zs}\left(h\right)\right)-\left(\sigma_{yc}\left(h\right)-\sigma_{zc}\left(h\right)\right)}{2}\Delta l \end{array}$$

Finally, it can be deduced that if we want to achieve higher OVS measurement accuracy without being affected by the external stress, the external stress should satisfy the following equation:

(4) $$\sum_{h=1}^{q}\left[\left(\sigma_{ys}\left(h\right)-\sigma_{zs}\left(h\right)\right)-\left(\sigma_{yc}\left(h\right)-\sigma_{zc}\left(h\right)\right)\right]\cdot \Delta l=0$$

i.e., the spatial distribution of (*σ_y_* − *σ_z_*) in the dual-crystal should be symmetrical.

**Figure 4:**
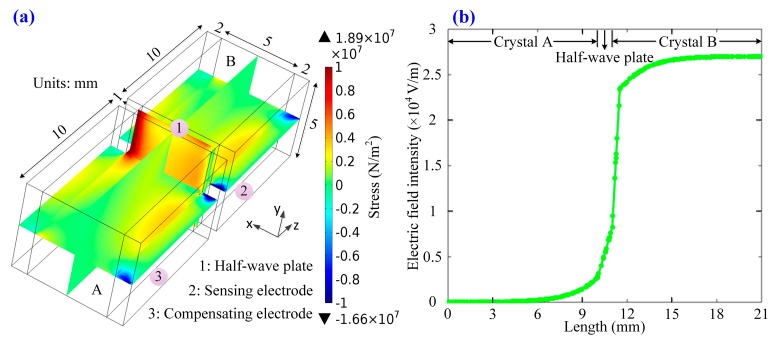
(**a**) The distribution of (*σ_y_* − *σ_z_*) in the designed dual-crystal sensing unit; (**b**) the distribution of electric field when the electrodes of the crystal B is applied with a voltage 1 kV.

The simulation results about the distribution of (*σ_y_* − *σ_z_*) are described in Figure 3. It is noted that the temperature variation may destroy the symmetry of the distribution of (*σ_y_* − *σ_z_*) in the two crystals when the (110) surface of crystal is fixed. However, if the (001) surface of crystal is fixed on the mechanical device, the minimum absolute value and good distribution symmetry of (*σ_y_* − *σ_z_*) are guaranteed in the two crystals. According to the above theoretical analysis of the key parameters of the OVS reciprocity degeneration, a novel structure of the dual-crystal sensing unit is designed, as shown in Figure 4a. Figure 4a shows the spatial distribution of stress (*σ_y_* − *σ_z_*) in the novel structure of the dual-crystal sensing unit. This compensation effect is nearly equivalent to *σ_ys_* = *σ_yc_*, *σ_zs_* = *σ_zc_*, so that OVS can effectively offset the unwanted stresses on the crystals caused by temperature variation. Then, we obtain that the ideal equivalent Pockels phase in crystal A and B: *δ* = π·*l*·*n*_0_^3^·*γ*_41_·*U*/(*λ*·*d*).

The electric field distribution along the light propagation path in the dual-crystal sensing unit is also simulated when a measured voltage of 1 kV is imposed on the electrodes of the crystal B as shown in Figure 4b. We can see that the novel structure of OVS guarantees the uniform distribution of the electric field in sensing crystal B, while the electric field of compensatory crystal A is nearly zero.

### 2.2. Error Analyses and Optimization of the Pockels Phase Transfer Unit

Based on the new sensing structure, the light transmission model can be established and the influence of the unideal optical parameters on the measurement accuracy of OVS can be investigated. The models of axis-angle errors are depicted in Figure 1, where the light transmission model in the OVS has the form:

(5) $$E(t)=B(t)\cdot Q\cdot D\cdot R\cdot D^{T}\cdot Q^{T}\cdot B^{T}(t-\tau )\cdot E_{SLD}$$

where *τ* is the transmitting time during the back and forth process, *B*(*t*) = *P*·*W*_1_·*M*(*t*)·*W*_2_, *D* = *V*·*C*·*H*·*S*, and *W*_1_, *W*_2_ and *V* satisfy $\left[\begin{array}{cc} \text{cos}\;\theta_{i} & \text{sin}\;\theta_{i}\\ -\text{sin}\;\theta_{i} & \text{cos}\;\theta_{i} \end{array}\right]$, *i* = 1, 2, 3, as described in Figure 1 and Figure 2. Therefore, the interference light intensity that the PIN-FET captures is:

(6) $$I_{out}(t)=|E^{*}(t)\cdot E(t)|$$

As only the 45° splice has unwanted welding angle error, the interference light intensity, based on Equation (6), can be rewritten as:

(7) $$I_{out}(t)=\frac{1}{4}\left\{\left(1-\text{cos}\;4\theta_{1}\right)\text{cos}\left[\varphi \left(t\right)-\varphi \left(t-\tau \right)-2\delta \right]\right\}$$

where *θ*_1_ is the actual welding angle of the 45° splice. In closed-loop detection, *φ*(t) − *φ*(t-*τ*) = *φ*_b_ + *φ_f_*, where *φ*_b_ = π/2 when *t* = (2*k* + 1)·τ and *φ*_b_ = −π/2 when *t* = 2*k*τ (*k* = 0, 1, 2, …), *φ_f_* is the closed-loop feedback phase, which is equivalent to Pockels phase 2*δ*. Then, we can have:

(8) $$I_{out}\left(t\right)=\frac{1}{4}\left\{\left(1-\text{cos}\;4\theta_{1}\right)\text{cos}\left[\varphi_{b}+\varphi_{f}-2\delta \right]\right\}=\mp \frac{1}{4}\left\{\left(1-\text{cos}\;4\theta_{1}\right)\text{sin}\left[\varphi_{f}-2\delta \right]\right\}$$

It is noted that *θ*_1_ only affects the interference intensity instead of the feedback phase and the interference light power fluctuation can be suppressed by using the closed-loop detection method [14,15]. Due to the two orthogonal linear-polarized beams generated after passing the 45° splice, the unideal welding angles before OPM have no influence on the feedback phase. Therefore, it is obtained that the unideal polarizer and 45° splice are not the key factors that affect the voltage sensor capability.

**Figure 5:**
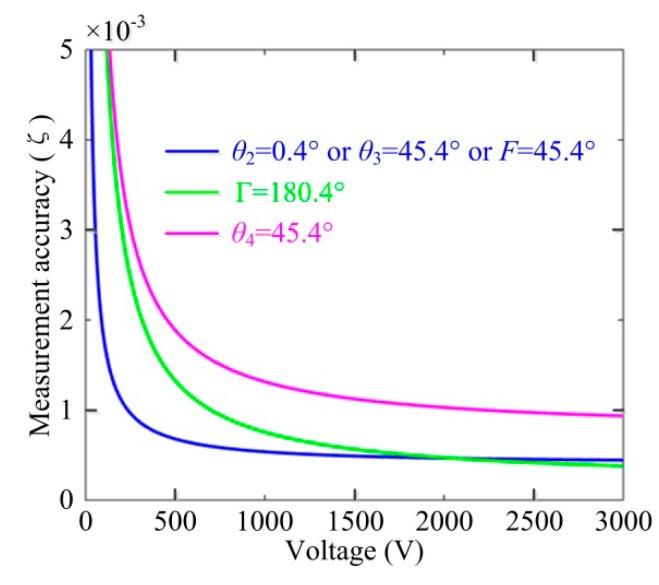
The impact of the optical device axis-angle errors on the OVS measurement accuracy.

Furthermore, the influence of the key axis-angle errors on the OVS working performance can be explored according to Equations (5) and (6), as well as the simulation platform of OVS. Supposing that one of *θ*_2_, *F*, *θ*_3_, Γ and *θ*_4_ is +0.4° while others are +0.1°, we can analyze the impact of these angle errors on the OVS measurement accuracy using the simulation platform shown in Figure 5. The relative measurement error of OVS can be expressed as *ζ* = |*φ_f_* − 2*δ*|/2*δ* × 100%. Note that the axis angle between the half-wave plate and BGO crystal, *θ*_4_, is the most significant factor that affects the measurement precision of OVS.

Now, a detailed theoretical analysis of the main axis-angle error of OVS can be conducted. Assuming that only *θ*_4_ has an angle error, one can obtain the interference light intensity based on Equation (6):

(9) $$\begin{array}{cc} I_{out}= & \frac{1}{4}\{1+\left(1+\text{cos}\;2\delta \right)\left(1-\text{cos}\;8\theta_{4}\right)/4\cdot \text{cos}\left[\varphi \left(t\right)-\varphi \left(t-\tau \right)\right]+\text{sin}\;2\delta \cdot {\text{cos}}^{2}\;2\theta_{4}\cdot \text{sin}\left[\varphi \left(t\right)-\varphi \left(t-\tau \right)\right]\\ & -\text{cos}\left[\varphi \left(t\right)-\varphi \left(t-\tau \right)-2\delta \right]\} \end{array}$$

where *θ*_4_ is the actual axis-angle between the half-wave plate and BGO crystal. With the similar deducing process of *θ*_1_ in the closed-loop detection, the influence of *θ*_4_ on the interference light power intensity can be rewritten as:

(10) $$\begin{array}{cc} I_{out}= & \frac{1}{4}[1+\text{sin}\;2\delta \cdot {\text{cos}}^{2}\;2\theta_{4}\cdot \text{sin}\left(\varphi_{b}+\varphi_{f}\right)+\left(1+\text{cos}\;2\delta \right)\left(1-\text{cos}\;8\theta_{4}\right)/4\cdot \text{cos}\left(\varphi_{b}+\varphi_{f}\right)\\ & -\text{cos}\left(\varphi_{b}+\varphi_{f}-2\delta \right)]\\ = & \frac{1}{4}\left\{1+\left({\text{cos}}^{2}\;2\theta_{4}-1\right)\text{sin}\;2\delta \cdot \text{sin}\left(\varphi_{b}+\varphi_{f}\right)+\left[\left(1+\text{cos}\;2\delta \right)\left(1-\text{cos}\;8\theta_{4}\right)/4-\text{cos}\;2\delta \right]\cdot \text{cos}\left(\varphi_{b}+\varphi_{f}\right)\right\}\\ = & \frac{1}{4}\left[1\mp \text{sin}\left(\varphi_{f}-2\delta_{u}\right)\right] \end{array}$$

where 2*δ_u_* is the function of 2*δ* and *θ*_4_. One can find that the relationship between the feedback phase *φ_f_* and 2*δ* becomes nonlinear due to the unideal *θ*_4_. The feedback phase of the real closed-loop detection system is used to offset 2*δ_u_*, which is relevant to 2*δ* and satisfies *φ_f_* − 2*δ_u_* = 0. Therefore, the practical feedback phase is given by:

(11) $$\varphi_{f}=-actg\frac{2\left(\text{cos}\;4\theta_{4}-1\right)\cdot \text{sin}\;2\delta }{\left(1+\text{cos}\;2\delta \right)\left(1-\text{cos}\;8\theta_{4}\right)-4\;\text{cos}\;2\delta }$$

**Figure 6:**
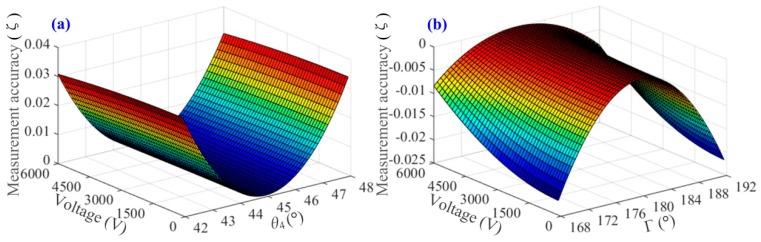
The relative measurement error of OVS when (**a**) the axis-angle between half-wave plate and BGO crystal *θ*_4_ is varying or (**b**) the phase delay angle of half-wave plate Γ is varying.

Moreover, the influence of the imperfect phase delay angle of half-wave plate Γ is similar to the unideal *θ*_4_. Thus, it is obtained that the unideal half-wave plate and the axis-angle error between the half-wave plate and BGO crystals will arouse the nonlinearity of OVS and that the unideal axis-angle will induce the relative measurement error of OVS, as depicted in Figure 6.

As a matter of fact, the axis-angle errors of the optical components are inevitable and it is also difficult to quantify them during the manufacturing process. However, with our simulation platform, we notice that the axis-angle errors between different optical components can aggravate or offset the influence on OVS working capability. The simulation results are shown in Table 1 and the measurement accuracy of OVS can be optimized by compensating the axis-angle errors based on these results. The mean is if *θ*_4_ is less or greater than the ideal value, the actual welding angle of the 0° splice (*θ*_2_), the actual rotation angle of FR (*F*) and the actual axis-angle between FR and the BGO crystal (*θ*_3_) should also be constructed less or greater than the ideal angle. In general, a better axis-angle compensation of our proposed configuration can be achieved with a smaller nonlinear OVS system.

In this work, the key parameters of the stress distribution symmetry are investigated in order to build a novel sensing device, which can counteract unwanted stresses of the two crystals caused by temperature variation. And, the angle-axis compensation method is proposed to neutralize unavoidable axis-angle errors of optical components in the manufacturing process for improving the linearity and measurement sensitivity of OVS. These novel methods improve the measurement precision of OVS under different temperature environment, which also promote the performance of OVS to the measurement of higher voltages in transmission lines.

## 3. Experiments

To demonstrate the validity and effectiveness of our proposed designs, several experiments were conducted and are described in this section.

**Figure 7:**
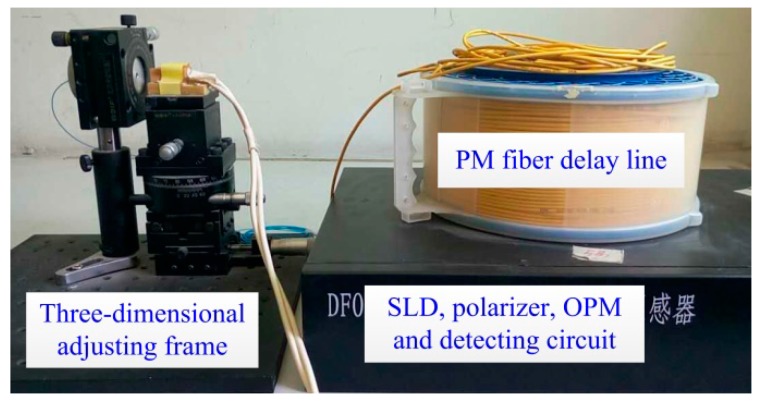
The experimental setup of FR axis-angle measurement.

**Figure 8:**
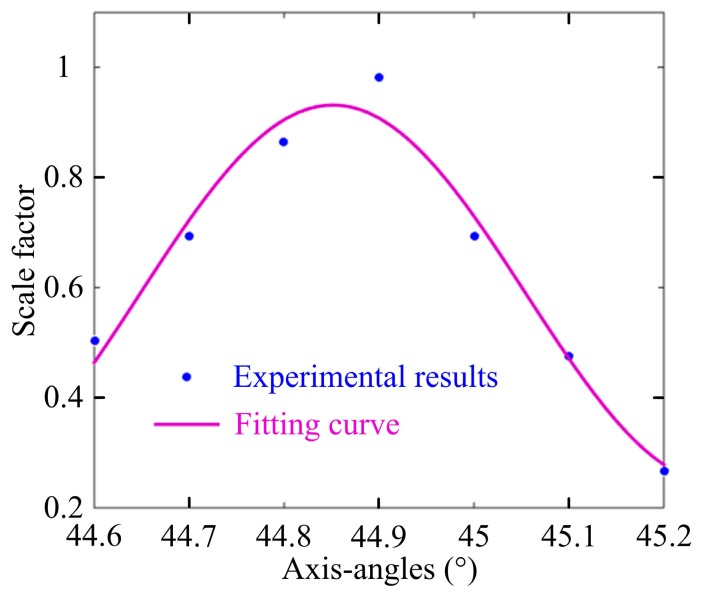
The tendency of scale factor with different axis-angles *θ*_3_.

As shown in Figure 7, the first experiment is carried out to validate the axis-angle compensation method during the manufacturing process of OVS. The FR is placed in a three-dimensional adjustment frame, and is adjusted to ensure that the output light beam shoots the center of the end surface of the BGO crystal. Then, the axis-angle between the FR and the BGO crystal is rotated by adjusting the three-dimensional adjustment frame, and the interference light intensity at PIN-FET is observed. By real-time monitoring of interference light intensity of OVS, we can rotate the actual axis-angle (*θ*_3_) between FR and the BGO crystal, so that axis-angle compensation of *θ*_3_ offsets the unideal axis-angle of the half-wave plate in the sensor head. Figure 8 shows the tendency of scale factor with different axis-angles *θ*_3_. In the practical experiment, the larger interference light intensity means the larger scale factor and the smaller nonlinearity of the sensor, which is consistent with the theoretical analysis given in this paper. 

The experimental phenomenon is more apparent with *θ*_3_ < 45° than *θ*_3_ > 45°, which indicates that the actual axis-angle (*θ*_3_) between FR and the BGO crystal can be used to offset the unideal axis-angle of the half-wave plate. And the OVS measurement error caused by the unideal axis-angle of the half-wave plate in the sensor head is better offset, when the axis-angle between FR and the BGO crystal (*θ*_3_) is rotated with angle −0.1° of 45°. This experiment verifies the effectiveness of our proposed angle error compensation method during the OVS manufacturing process. 

**Figure 9:**
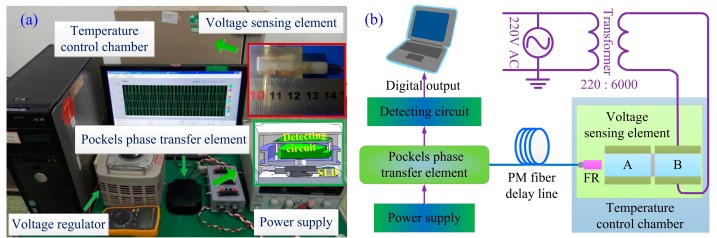
(**a**) Experimental OVS prototype; (**b**) schematic diagram of experiment.

The designed dual-crystal voltage sensing unit and the Pockels transfer unit of OVS are shown in Figure 9b. In the configuration of Pockels phase transfer unit, the SLD is fixed on the metal base for exothermic convenience with room temperature variation to suppress light wavelength drift. The dual-crystal sensing unit is independently designed using the configuration depicted in Figure 4a. The voltage sensing unit can be separated to measure the voltage by the PM fiber delay line, so that the Pockels phase transfer unit can be placed in the room to avoid environmental disturbance.

To further test and verify the measurement precision of OVS based on our proposed designs, we also carry out some experiments to test the performance of the novel OVS at room temperature or over a wide temperature variation of −40 °C~+60 °C. The applied voltage, in the range of 0~4000 V, is provided by a 220 V AC power supply and a transformer with the ratio 220:6000. The interference light intensity from the Pockels phase transfer unit is detected and processed by the detecting circuit, and finally, the data are sent to the computer.

**Figure 10:**
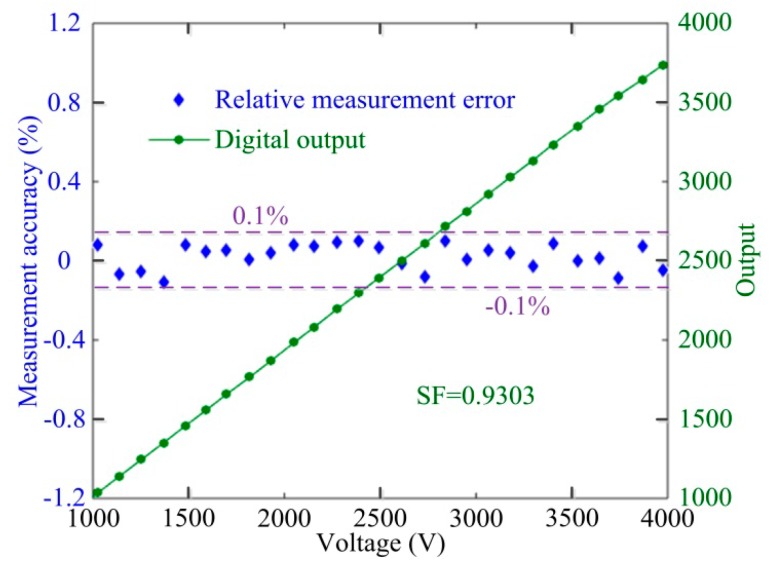
Relative measurement error, *SF* and digital output of OVS.

First, we conduct the scale factor (SF) experiments at room temperature. As shown in Figure 10, the relative measurement error of OVS, as calculated by solving *ζ* = |(*U*_out_/*SF* − *U*)/*U* × 100%| with digital output *U*_out_, is less than ±0.1% when the applied voltages are over 1000 V. There is good linearity between the digital output and the input voltage, which also verifies that the proposed axis-angle compensation method is effective and the OVS measurement accuracy is therefore improved. Thus, the axis-angle compensation method can neutralize unavoidable axis-angle errors of optical components in the manufacturing process for optimizing the measurement precision of OVS.

**Figure 11:**
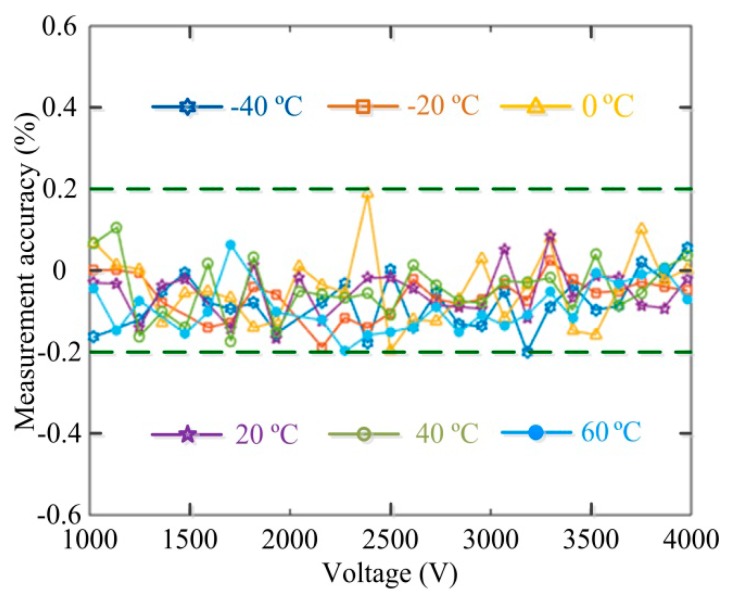
Relative measurement errors of OVS at different temperatures.

Further experiments are conducted to test the temperature stability in the temperature range from −40 °C~+60 °C. In these experiments, the dual-voltage sensing unit of OVS is put into a temperature-controlled chamber and the environmental temperature variation is monitored by temperature sensor. In the temperature dependence experiment, we measure the detection error of OVS for each temperature point with a temperature difference of 20 °C between adjacent point during −40 °C~+60 °C, and the experimental method is the same as that reported in [18]. The measurement accuracy of OVS within the temperature range of −40 °C~+60 °C is obtained as shown in Figure 11. 

**Figure 12:**
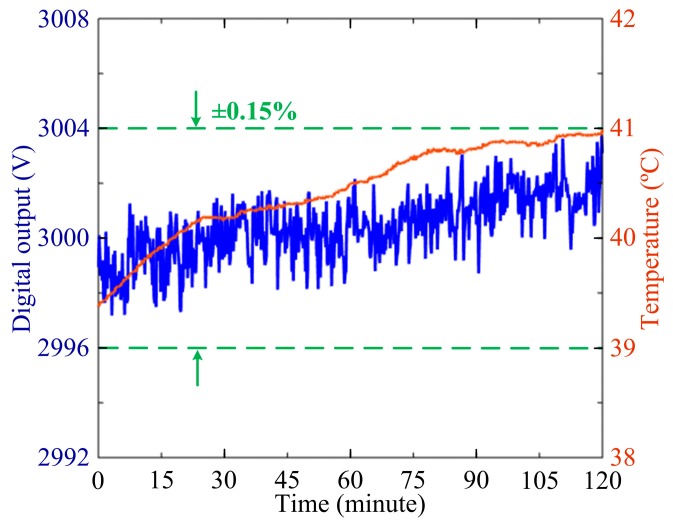
The long-term measured stability of OVS at 3000 V AC over two hours when the temperature is maintained at +40 °C by the temperature-controlled chamber.

The long term measure stability of the sensor at 3000 V AC over two hours is also measured when the temperature is maintained at +40 °C by the temperature-controlled chamber as shown in Figure 12. The relative measurement errors of OVS are less than ±0.2% in the temperature range of −40 °C~+60 °C, which is much improved as compared with the value ±0.3% in the range of 0 °C~+60 °C reported in [18]. It is obvious that the temperature stability is greatly improved. The experimental results verify that the measurement accuracy of OVS satisfies the demand of IEC 60044-7 for 0.2-level measurement [20]. In particular, the novel sensing scheme could be applied in the engineering system of OVS to improve the performance of the sensor in a smart grid.

## 4. Conclusions

The varying temperature field in practical engineering and the unwanted axis-angle errors between optical components can induce OVS system reciprocity degradation and further hinder the application of dual-crystal OVS in a smart grid. In this work, the inducing principle of OVS reciprocity degradation is analyzed and the optimized method to guarantee the system reciprocity is proposed. Firstly, the linear-polarized light propagation model of the dual-crystal sensing unit is established and the key parameters of the OVS reciprocity degeneration are investigated by taking the applied electric, temperature and stress fields into consideration. Secondly, for the Pockels phase transfer unit, the influencing principle of the axis-angle errors on the nonlinearity between the OVS input and output are also analyzed. Finally, the compensation method of axis-angle errors is proposed to improve the measurement accuracy of OVS. The relative measurement errors ±0.2% over a temperature range of −40 ºC~+60 ºC are successfully demonstrated in the reciprocal dual-crystal sensing scheme of OVS. Such relative measurement errors of the novel OVS under the influence of temperature variation meet the requirement of IEC 60044-7 for 0.2-level measurement. Moreover, the miniaturized structures of optical components in our designed configurations are of significance for expanding the application of OVS in a smart grid.

## Figures and Tables

**Table 1:** Simulation results about the angle-axis compensation method of angle errors.

*θ*_2_ (°)	*F* (°)	*θ*_3_ (°)	Г (°)	*θ*_4_ (°)	Minimum Voltage within 0.1% Accuracy (V)
0.2	45.2	45.2	179.8	45.2	356
−0.2	45.2	45.2	179.8	44.8	593
−0.2	45.2	44.8	179.8	44.8	744
−0.2	44.8	44.8	179.8	44.8	1517
−0.2	44.8	44.8	180.2	44.8	356
0.2	44.8	44.8	180.2	45.2	593
0.2	44.8	45.2	180.2	45.2	744
0.2	45.2	45.2	180.2	45.2	1517

## Appendix A

In this appendix, more detailed information about how to solve the propagation model of the electromagnetic waves in the voltage sensing unit is provided, which is previously described in Section 2.1. Considering the influences of the Pockels effect, thermo-optic effect and elastic-optic effect, the dielectric tensor of the BGO crystal changes to:

(A1) $$[\varepsilon ]=\varepsilon_{0}\cdot \{[\varepsilon^{0}]+[\Delta \varepsilon_{P}]+[\Delta \varepsilon_{T}]+[\Delta \varepsilon_{S}]\}=\left[\begin{array}{ccc} \varepsilon_{xx} & \varepsilon_{xy} & \varepsilon_{xz}\\ \varepsilon_{yx} & \varepsilon_{yy} & \varepsilon_{yz}\\ \varepsilon_{zx} & \varepsilon_{zy} & \varepsilon_{zz} \end{array}\right]$$

Substituting Equation (A1) into Equation (1), we have [9]:

(A2) $$k_{1,2}^{2}=\frac{\omega^{2}\mu }{2}\left[(\varepsilon_{xx}+\varepsilon_{yy})\pm \sqrt{{\left(\varepsilon_{xx}-\varepsilon_{yy}\right)}^{2}+4\varepsilon_{xy}\varepsilon_{yx}}\right]$$

where *k*_1_ and *k*_2_ are the two possible solutions of the propagation constant. Then, the equivalent Jones matrix of each segment Δ*l* of the crystal is $C=\left[\begin{array}{cc} a & b\\ c & d \end{array}\right]$, where [9]:

(A3) $$\begin{array}{l} a=\text{cos}\;\phi -j\left[\left(\varepsilon_{yy}-\varepsilon_{xx}\right)/\sqrt{{\left(\varepsilon_{yy}-\varepsilon_{xx}\right)}^{2}+4\varepsilon_{xy}\varepsilon_{yx}}\right]\text{sin}\;\phi =d^{*},\\ b=\left(2j\varepsilon_{xy}/\sqrt{{\left(\varepsilon_{yy}-\varepsilon_{xx}\right)}^{2}+4\varepsilon_{xy}\varepsilon_{yx}}\right)\text{sin}\;\phi ,\\ c=\left(2j\varepsilon_{yx}/\sqrt{{\left(\varepsilon_{yy}-\varepsilon_{xx}\right)}^{2}+4\varepsilon_{xy}\varepsilon_{yx}}\right)\text{sin}\;\phi ,\\ \phi =\left(k_{1}-k_{2}\right)\cdot \Delta l/2 \end{array}$$

Moreover, in order to describe the reciprocity degeneration of the voltage sensing unit, one can rewrite Equation (A2) as:

(A4) $${\left(k_{1}-k_{2}\right)}^{2}=\omega^{2}\mu \left\{{\left(\sqrt{\varepsilon_{xx}}-\sqrt{\varepsilon_{yy}}\right)}^{2}+2\sqrt{\varepsilon_{xx}\varepsilon_{yy}}\left[1-\sqrt{1-\left(\varepsilon_{xy}\varepsilon_{yx}\right)/\left(\varepsilon_{xx}\varepsilon_{yy}\right)}\right]\right\}$$

Noting that the intrinsic parameter relations of the BGO crystal, *ε_xy_,ε_yx_* << *ε_xx_,ε_yy_*, we can approximately consider the ratio (*ε_xy_ε_yx_*)/(*ε_xx_ε_yy_*) as an infinitesimal. Then, Equation (A4) is simplified into:

(A5) $${\left(k_{1}-k_{2}\right)}^{2}=\omega^{2}\mu \left[{\left(\sqrt{\varepsilon_{xx}}-\sqrt{\varepsilon_{yy}}\right)}^{2}+\left(\varepsilon_{xy}\varepsilon_{yx}\right)/\sqrt{\varepsilon_{xx}\varepsilon_{yy}}\right]$$

as *ε_xy_* = *ε_yx_* = Δ*ε_xy_* = *Δε_yx_*. At the same time, the inequalities Δ*ε_xx_* << *ε_r_*, Δ*ε_yy_* << *ε_r_*, (Δ*ε_xx_*+Δ*ε_yy_*)/*ε_r_* << (Δ*ε_xy_Δε_yx_*)^2^ and (Δ*ε_xx_*Δ*ε_yy_*)/*ε_r_*^2^ << (Δ*ε_xy_Δε_yx_*)^2^ hold true. By repeatedly applying the Taylor series expansions of infinitesimals to Equation (A5), we obtain:

(A6) $${\left(k_{1}-k_{2}\right)}^{2}=\frac{\omega^{2}\mu \varepsilon_{0}}{4\varepsilon_{r}}\left[{\left(\Delta \varepsilon_{xx}-\Delta \varepsilon_{yy}\right)}^{2}+4\varepsilon_{xy}\varepsilon_{yx}\right]$$

and substituting Δ*ε_xx_* = −*ε_r_*^2^Δ*β*_1_, Δ*ε_yy_* = −*ε_r_*^2^Δ*β*_2_, Δ*ε_xy_* = −*ε_r_*^2^Δ*β*_6_ and Δ*ε_yx_* = −*ε_r_*^2^Δ*β*_6_ into Equation (A6), we have:

(A7) $${\left(k_{1}-k_{2}\right)}^{2}=\omega^{2}\mu \varepsilon_{0}\varepsilon_{r}^{3}\left[{\left(p_{11}-p_{12}\right)}^{2}\tau_{yz}^{2}+{\left(\gamma_{41}\cdot E+p_{44}\cdot \frac{\sigma_{y}-\sigma_{z}}{2}\right)}^{2}\right]$$
